# Cost-utility analysis of home treatment versus inpatient treatment in child and adolescent psychiatry: a decision analysis

**DOI:** 10.3389/frcha.2026.1923381

**Published:** 2026-08-10

**Authors:** Karolina Foremnik, Gaby Sroczynski, Barbara Buchberger

**Affiliations:** 1 Medical Faculty, University of Duisburg-Essen, Essen, Germany; 2 Institute of Public Health, Medical Decision Making and Health Technology Assessment, UMIT TIROL - University for Health Sciences and Technology, Hall in Tirol, Austria; 3 Robert Koch Institute, Berlin, Germany

**Keywords:** acute psychiatric crisis, child and adolescent psychiatry, cost-utility analysis, home treatment, inpatient treatment, Markov model

## Abstract

**Introduction:**

Health-economic evidence comparing home treatment (HT) and inpatient treatment (IT) in child and adolescent psychiatry is scarce. This study assessed the cost-effectiveness of both interventions for children and adolescents in acute psychiatric crises using a decision analysis.

**Methods:**

We developed a Markov state-transition model to evaluate the cost-effectiveness of HT compared to IT in children and adolescents with an acute psychiatric crisis, over a 3-year time horizon from a German healthcare provider perspective. Evaluated outcomes included quality-adjusted life years (QALYs) gained, total costs, and the incremental cost-utility ratio (ICUR; in Euro per QALY). Transition probabilities and utility values were obtained from the published literature. Direct medical and direct non-medical costs for IT were derived from German reference data published by the Institute for the Hospital Remuneration System (InEK). Costs for HT were estimated using micro-costing based on the German ward-equivalent treatment model (Stationsäquivalente Behandlung, StäB). Costs and effects were discounted at 3% annually. One-way and probabilistic sensitivity analyses assessed the robustness of the base-case analysis results. Results were reported in accordance with the Consolidated Health Economic Evaluation Reporting Standards (CHEERS) 2022 checklist.

**Results:**

HT was associated with lower discounted costs (61,213 €) and slightly fewer discounted QALYs (1.801 QALYs) compared to IT (total costs: 93,737 € and 1.807 QALYs). Moving from HT to IT was associated with 0.006 QALYs gained and resulted in an ICUR of 5,417,376 €/QALY gained. Results were robust in sensitivity analyses over a wide range of parameter variation.

**Discussion:**

This cost-utility analysis suggests that HT may be a cost-effective alternative to IT in children and adolescents with acute psychiatric crises in Germany, primarily driven by substantial cost savings during the acute phase. However, due to limited and heterogeneous clinical data, results should be interpreted cautiously. Future randomized studies with longer follow-up and child and disease-specific quality-of-life measures are needed to validate these findings.

## 1 Introduction

Mental health conditions are a leading cause of disease burden among young people worldwide, affecting approximately 14% of children and adolescents aged 10–19 (1). This global crisis was compounded by the COVID-19 pandemic: international multi-center data from 62 emergency departments across 25 countries revealed that psychiatric emergencies among adolescents increased by 50% during the pandemic compared with pre-pandemic levels (2).

Despite this growing demand, access to treatment remains constrained (1, 3). Structural and individual barriers—such as waiting times or fear of stigmatization—substantially limit timely access to treatment for children and adolescents (3). Inpatient treatment (IT) is traditionally considered the standard treatment for children and adolescents experiencing severe acute psychiatric crises, but primarily focuses on symptom stabilization and requires temporary separation from the family and everyday environment (4, 5). Moreover, because the inpatient setting provides a highly structured and controlled environment, improvements achieved during inpatient treatment may not be maintained once patients return to a home environment that is often more complex and, in some cases, dysfunctional (4).

These conceptual and capacity-related limitations have driven the development of HT-based strategies as an alternative for managing acute psychiatric crises within the patient's home environment (6). HT is commonly used as an umbrella term for a heterogeneous group of outreach-based interventions; in this study, it refers specifically to outreach-based treatment delivered at home as a clinically viable alternative to inpatient admission (6, 7). In Germany, HT for children and adolescents is formally regulated under the German operation and procedure code (OPS 9–801) as stationsäquivalente Behandlung (StäB, ward-equivalent home treatment)—a standardized model with defined structural requirements, including daily patient contact, weekly multiprofessional team meetings, and 24/7 crisis availability (8). Embedded in the national hospital reimbursement system (PEPP), this framework provides a transparent and reproducible basis for economically modeling the otherwise heterogeneous concept of HT.

Although HT is considered a promising alternative to inpatient acute treatment in child and adolescent psychiatry (9, 10), evidence on its cost-effectiveness remains limited (9). Available economic evaluations suggest potential cost-effectiveness of sequential HT approaches: Boege et al. reported that HoT-BITs (Hometreatment brings inpatient-treatment outside) may be dominant, offering lower costs alongside with superior clinical outcomes (11), while Ougrin et al. found the Supported Discharge Service (SDS) to be cost-effective compared with usual treatment (12).

Model-based evaluations in child and adolescent psychiatry remain rare, particularly those assessing patient-relevant outcomes over a longer time horizon: a recent systematic review identified only 12 relevant studies, none of which modeled home treatment vs. inpatient treatment (13). The aim of this study was therefore to provide the first Markov model-based cost-utility analysis comparing home treatment with inpatient treatment for children and adolescents experiencing acute psychiatric crises, to inform evidence-based service planning and resource allocation decisions in child and adolescent treatment under conditions of limited resources.

## 2 Materials and methods

### 2.1 Model structure

**Figure 1:**
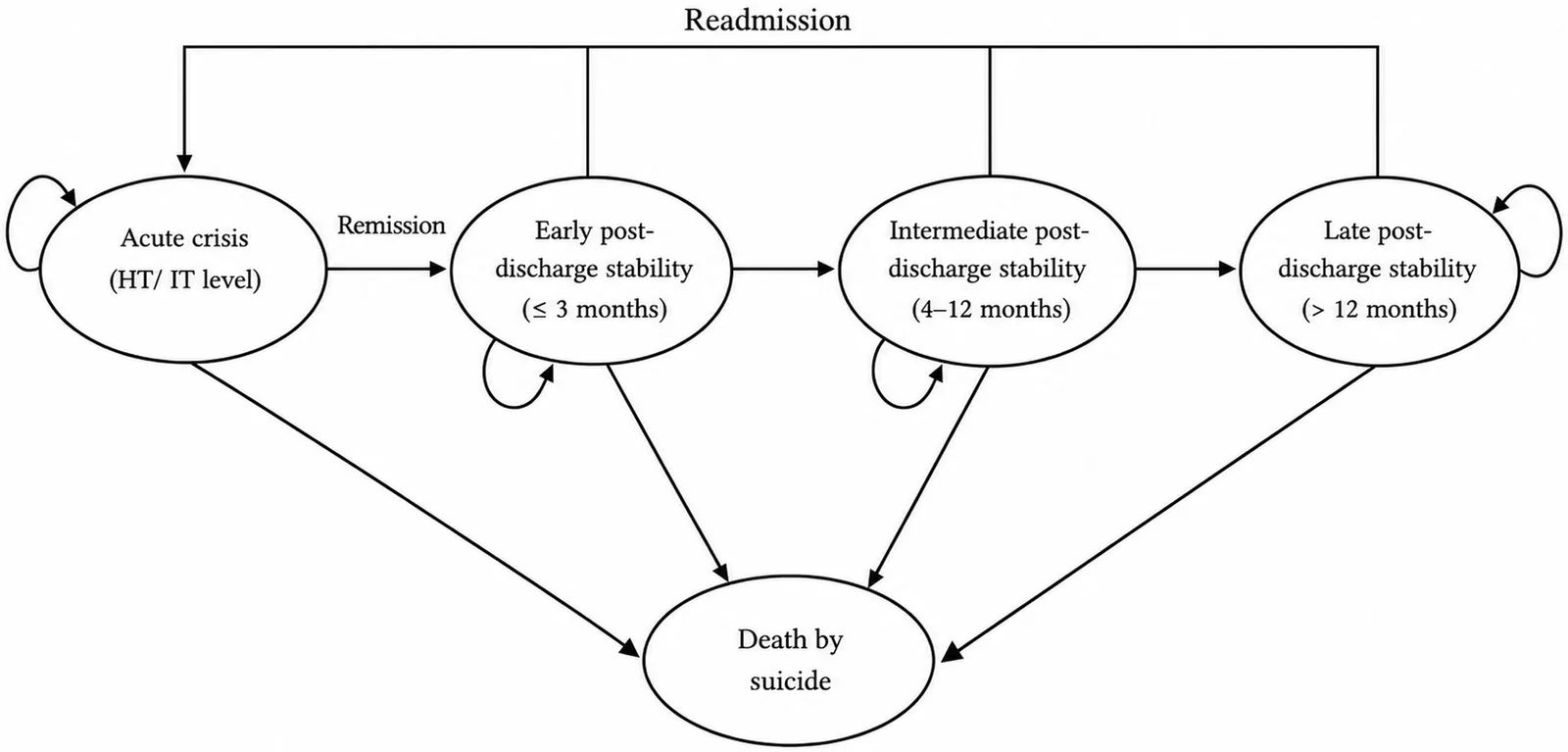
Model structure.

A Markov state-transition model with a cohort simulation was developed in accordance with the ISPOR-SMDM modeling guidelines (14) to simulate the course of potentially recurrent episodes of acute crisis over time and the clinical and health-economic consequences of the applied interventions. The model comprised five discrete Markov states (Figure 1): acute crisis (requiring either home treatment or inpatient treatment), early post-discharge stability (0–3 months), intermediate post-discharge stability (4–12 months), late post-discharge stability (>12 months), and death by suicide. A time horizon of three years was applied, informed by the available follow-up data (10). A lifetime horizon was not considered appropriate, as the treatment alternatives were not evaluated with respect to long-term mortality outcomes (14). The cycle length was set to one month to adequately capture the acute course and short-term dynamics of psychiatric crises in children and adolescents (14).

The initial cohort entered the model in the health state “acute crisis”, representing a level of psychiatric symptom severity requiring intensive treatment, delivered either as IT or HT. From the acute crisis state, individuals could either remain in acute crisis or transition to the state “early post-discharge stability”, representing remission within the first three months following discharge. In this model, remission was defined as a clinical condition in which hospital-equivalent psychiatric treatment was no longer required. Clinical stabilization sufficient for discharge was assumed, although outpatient follow-up treatment could continue. After completion of the early post-discharge phase, individuals may transition to the intermediate stability state (4–12 months) and subsequently to the late stability state (>12 months post discharge). In all post-discharge states, individuals are at risk of readmission, resulting in recurrence of an acute psychiatric crisis. The subdivision of the post-discharge period into distinct time phases reflects the elevated risk of readmission following discharge, which decreases over time (15, 16).

In all health states, individuals were assumed to be at risk of suicide-related death. Other-cause mortality was not considered, due to the relatively short time horizon of the analysis and low other-cause mortality in the target population.

### 2.2 Analytical framework

The analysis followed a cost-utility framework comparing HT with IT. Results are reported in accordance with the Consolidated Health Economic Evaluation Reporting Standards (CHEERS) 2022 checklist (17).

The target population comprised children and adolescents under the age of 18 experiencing acute psychiatric crises requiring hospital-equivalent treatment. No restrictions were applied regarding socioeconomic or cultural background. Participants had to present with severe mental disorders with marked functional impairment (first episode or as an acute exacerbation); comorbidities were not exclusionary. Studies addressing acute mental health crises such as suicidality, self-harm, mood or anxiety disorders, psychosis, agitation, or trauma-related symptoms were eligible. Exclusion criteria included chronic psychiatric courses requiring long-term, community-based treatment (e.g., Assertive Community Treatment) and severe somatic conditions (e.g., intoxication states or withdrawal, anorexia-related decompensation). Neurodevelopmental disorders and intellectual disabilities were not exclusionary when present as comorbid conditions. However, studies were excluded when these disorders were the primary condition, and the intervention was specifically designed for their long-term management rather than for the treatment of an acute psychiatric crisis.

This study did not use an original patient-level dataset. Demographic and clinical characteristics were therefore available only as reported in the respective source publications. Treatment duration and cumulative readmission proportions were obtained from Graf et al. (18); (mean age 15.9 years, 48%–69% female, predominantly affective, anxiety, and behavioral disorders) (18); health-state utilities were derived from Ougrin et al. (12); (mean age 16.2–16.3 years, 62%–68% female, unrestricted diagnostic spectrum of psychiatric emergencies) (12); and post-discharge suicide mortality was informed by Chung et al. (19); (heterogeneous diagnoses including psychosis, pooled across female-only, male-only, and mixed-sex samples) (19). As no consistent age-, sex-, or diagnosis-specific parameter set was available across all three sources, the modeled cohort was not stratified by age, sex, or diagnosis; a fixed entry age of 16 years was assumed, reflecting the mean ages reported by Graf et al. and Ougrin et al. Further demographic and clinical characteristics of the source populations are summarized in Supplementary Material.

Home treatment was defined as outreach-based, multidisciplinary psychiatric care delivered in non-clinical settings-such as the patient's home or school- as a direct clinical alternative to inpatient admission. Suitability for HT, including the safety of the home environment and caregiver availability, was generally assessed by the clinical team before treatment initiation. Eligible interventions required round-the-clock crisis support capacity and involvement of multiprofessional teams. Two treatment approaches can be distinguished: stand-alone home treatment, in which treatment is delivered entirely within the patient's home environment as a direct substitute for inpatient admission, and sequential inpatient-home treatment approaches, in which an initial period of inpatient stabilization is followed by home-based continuation of treatment (10). In the present analysis, the HT arm was operationalized as stand-alone home treatment for the estimation of transition probabilities and costs. However, no utility data specific for stand-alone HT were identified in the literature; utility values were therefore derived from Ougrin et al., who evaluated a supported discharge service reflecting a sequential approach (12).

Interventions were excluded if they were primarily designed as single-provider models, institutional programs (e.g., group homes or residential treatment facilities), preventive services, or community-based rehabilitation programs such as Assertive Community Treatment (ACT), as these primarily target chronic mental health conditions rather than acute crisis management. Interventions could range from short-term crisis stabilization to more extended treatment phases, reflecting the heterogeneity of real-world home treatment programs.

The comparator, standard inpatient psychiatric treatment, was defined as continuous 24/7 supervision and structured multidisciplinary treatment in a hospital-based setting. Studies with comparison groups located in emergency departments or general somatic hospital wards without structured psychiatric treatment were not considered. Partial hospitalization and day-hospital settings were also excluded as comparators, as these do not provide 24/7 supervision. Similarly, institutional residential treatment formats were excluded, as these typically represent longer-term therapeutic placements aimed at psychosocial rehabilitation rather than acute crisis management.

The primary outcomes were total undiscounted and discounted costs and quality-adjusted life years (QALYs), and the discounted incremental cost-utility ratio (ICUR), expressed as € per QALY gained. In the absence of an officially defined Willingness-to -Pay (WTP) threshold in Germany (20), a reference value of approximately 90,000 €/QALY was applied (21).

The analysis was conducted from the perspective of the German healthcare provider, considering direct medical and direct non-medical costs. For inpatient treatment, these were derived from full-cost data published by the German Institute for the Hospital Remuneration System (InEK). Corresponding costs for home treatment were estimated using a micro-costing approach based on the German ward-equivalent treatment model (Stationsäquivalente Behandlung, StäB). Germany serves as a reference setting for this analysis, as home treatment is formally regulated through the Operation and Procedure Classification catalogue (Operationen- und Prozedurenschlüssel, OPS) as StäB, with clearly defined structural requirements including daily patient contact (8).

Both, costs (in €) and QALYs were discounted at an annual rate of 3% (22). A half-cycle correction was applied (23). All model calculations and simulations were performed using AMUA Version 1.0 (1.8.0_491) (24) and Microsoft Excel. The model file is provided as Supplementary Material. Figures and graphical visualizations were created using RStudio Version 2026.01.0 + 392 and Excel (24, 25). Key model parameters are summarized in Table 1. Generative AI tools (ChatGPT (GPT-5.5 Thinking), Claude (Sonnet 4.6)) were used for language editing, wording refinement, and support with R coding. All content was reviewed, verified, and approved by the authors.

**Table 1:** Model parameters: transition probabilities, utilities, costs in € per month, and probabilistic distributions used in the base case, deterministic sensitivity analysis (DSA), and probabilistic sensitivity analysis (PSA).

Model parameter	Baseline	DSA Low	DSA High	PSA Distribution	Alpha (shape)	Beta (scale)
Transition probabilities (per month)						
Remission (acute → early)						
Remission HT	0.302	0.273	0.339	Beta	222.98	514.83
Remission IT	0.282	0.245	0.333	Beta	112.77	286.84
Readmission (post → acute)						
Readmission HT early	0.101	0.064	0.101	Beta	102.84	915.34
Readmission HT intermediate	0.028	0.018	0.028	Beta	117.07	4,048.08
Readmission HT late	0.008	0.005	0.008	Beta	113.12	13,922.00
Readmission IT early	0.069	0.045	0.069	Beta	120.18	1,629.16
Readmission IT intermediate	0.019	0.013	0.019	Beta	141.41	7,185.57
Readmission IT late	0.006	0.004	0.006	Beta	205.46	37,149.27
Suicide						
Suicide HT_IT acute^b^	0.00000267	—	—	—	—	—
Suicide HT IT early	0.0003079	0.000218	0.000438	Beta	30.89	97,693.00
Suicide HT IT intermediate	0.0001779	0.000126	0.000253	Beta	30.15	169,427.36
Suicide HT IT late	0.0001344	0.000095	0.000191	Beta	30.11	224,032.00
Utilities by state						
Utility baseline HT IT^a^	0.550	0.495	0.605	Beta	172.32	140.99
Utility post HT IT^a^	0.665	0.599	0.732	Beta	128.03	64.50
Costs in € per month						
Inpatient treatment						
Costs acute IT	17,765	15,405	22,467	Gamma	1,709.87	10.39
Home treatment (total)						
Costs acute HT^b^^c^	9,860	6,732	14,879	—	—	—
Home treatment (components)						
Costs direct treatment HT	2,041	1,257	3,837	Gamma	12.35	165.36
Costs fixed base HT	1,179	1,116	1,328	Gamma	509.14	2.32
Costs non patient facing time HT	1,075	315	2,744	Gamma	3.28	327.37
Costs travel personnel HT	2,668	1,868	3,469	Gamma	41.00	65.07
Costs travel operating HT	589	412	883	Gamma	26.46	22.26
Costs medical consumables HT^b^	211	—	—	—	—	—
Costs overhead HT	2,096	1,553	2,407	Gamma	80.01	26.20
Post-discharge (both arms)						
Costs post^b^	251	—	—	—	—	—

DSA, deterministic sensitivity analysis; PSA, probabilistic sensitivity analysis; HT, home treatment; IT, inpatient treatment; p, probability; u, utility; c, cost; rem, remission; readm, readmission; suic, suicide; early, early post-discharge phase (0–3 months); int, intermediate post-discharge phase (4–12 months); late, late post-discharge phase (>12 months); post, post-discharge.

a Utility values were equated across treatment arms in the base case; identical distributions were applied to HT and IT.

b Parameters marked with “—” were not varied in sensitivity analyses due to absence of reliable uncertainty estimates.

c c_acute_HT represents the sum of all home treatment cost components; individual components were varied separately in DSA and PSA. All costs are expressed in Euro (€) at 2025 price levels.

### 2.3 Transition probabilities

Readmission rates and follow-up durations varied substantially across the literature (ranging from 3.5 months to 4.3 years) (4, 12, 26, 27). Alternative sources did not report readmission consistently for both treatment arms, and follow-up periods were too short to capture medium-term readmission risk (4, 26–28). Baseline cumulative readmission probabilities were therefore extracted from Graf et al. reporting a cumulative readmission rate of 59.3% for HT and 41.7% for the IT over an 18–24-months follow-up period (18). These probabilities are higher than those generally reported in the broader pediatric psychiatric readmission literature, where cumulative readmission proportions after discharge range from 38% at one year to 43% at 30 months (15, 29, 30). However, these studies predominantly reflect mixed-intervention cohorts discharged into more restrictive post-discharge settings (e.g., residential or intensive community-based care), limiting their direct comparability to the present HT/IT framework.

Informed by the Kaplan–Meier survival curve from Joyce et al. (29), we distributed readmission events across time-dependent post-discharge health states as follows: 46.2% to the early phase (0–3 months post-discharge), 38.5% to the intermediate phase (4–12 months), and 15.4% to the late phase (>12 months). This decaying risk structure aligns with broader epidemiological data indicating that approximately 50% of pediatric psychiatric readmissions occur within the first quarter following discharge (15, 29–33). The monthly transition probabilities are detailed in Table 1. For HT, the monthly readmission probabilities were 0.101 (early), 0.028 (intermediate), and 0.008 (late); for IT, the corresponding values were 0.069, 0.019, and 0.006, respectively. Remission probabilities from the acute crisis state were calculated based on the mean lengths of stay reported by Graf et al. (18), yielding monthly transition probabilities of 0.302 for HT and 0.282 for IT.

Suicide mortality was operationalized across three time-dependent post-discharge phases (early, intermediate, late), with baseline probabilities derived from Chung et al. (19) and scaled to the pediatric/adolescent rate (Table 1). Given the absence of differential outcome data, monthly transition probabilities were assumed equal across HT and IT in all phases (Table 1). For the acute phase, in-hospital suicide risk was parametrized using Joint Commission data (3.2 per 100,000 admissions) (34), with an identical probability assumed for both treatment arms due to a lack of comparative data in child and adolescent psychiatry.

### 2.4 Measurement of utilities

Health state utility values were derived from Ougrin et al., the only identified study reporting EQ-5D-3L scores in a directly comparable population (supported discharge service vs. inpatient treatment, assessed at baseline and six months post-randomization) (12). As no statistically significant difference between groups was observed at either time point, utility values were averaged across both treatment arms and applied equally to HT and IT in the base case, yielding values of 0.55 for the acute crisis state and 0.665 for all post-discharge states per month (early, intermediate, and late). The utility for death by suicide was set to zero. QALYs were discounted at 3% per year (22). See Table 1.

### 2.5 Measurement of costs

For each health state, average monthly costs were assigned. Costs were estimated from the healthcare provider perspective and included direct medical costs associated with both inpatient and home treatment, as well as outpatient follow-up costs after discharge. Indirect costs (e.g., productivity losses of caregivers) were not included in the analysis. All costs were expressed as average costs per cycle in Euro (€) using the index year 2025. Historical personnel costs for home treatment were inflated to the 2025 price level using the German price index for health services (35). Costs were discounted at an annual rate of 3% (22). A half-cycle correction was applied (23).

Inpatient treatment costs were estimated using empirically derived full-cost data from the German Institute for the Hospital Remuneration System (InEK) for child and adolescent psychiatry (36). These data are based on real-world hospital cost calculations routinely reported by German hospitals participating in the national reimbursement system. Cost categories included personnel costs, pharmaceutical and medical supply costs, as well as medical and non-medical infrastructure costs (37). Average daily inpatient costs were calculated across child and adolescent psychiatric PEPP categories, resulting in estimated daily inpatient costs of 584€ per case. As the model aimed to reflect average treatment costs at the population level, aggregated mean costs across PEPP groups were used.

As the costs of home treatment are dependent on service characteristics such as treatment duration, contact frequency or team composition, a standardized reference framework was required for economic modeling. Therefore, the German model of StäB was used as a standardized reference framework (8). The requirements include daily patient contacts, weekly multiprofessional team meetings involving at least three professional groups, and weekly medical consultations. The provision of StäB on weekends and public holidays was explicitly accounted for in the cost calculations (8, 38).The OPS 9–801 requirement for weekday team availability was covered within standard working hours, while the required 24/7 medical intervention capability was assumed to be provided by the inpatient clinic and was not costed separately (8).

As no standardized cost data for StäB are available in the InEK cost data (39), full costs per treatment day were estimated using a resource-based bottom-up (micro-costing) approach. The objective was to capture the relevant InEK cost categories to ensure the highest possible comparability with the inpatient cost structure. A detailed description of the cost calculations is provided in the Supplementary Material.

Average treatment intensity and profession-specific treatment times for child and adolescent psychiatry were derived from the national evaluation report on StäB, based on German §21 Krankenhausentgeltgesetz (KHEntgG) routine data and OPS-coded treatment documentation (40). For the estimation of personnel costs related to direct patient treatment, average treatment minutes per day were obtained separately for each professional group and multiplied by the corresponding personnel costs per minute. Historical personnel costs from 2018 were inflated to the 2025 price level using the German price index for health services (35).

To account for additional indirect personnel time (e.g., documentation, handovers, coordination, and contacts with schools and youth welfare services) beyond direct patient contact, a conservative multiplier of 0.33 was applied, reflecting the ratio of direct contact time to total working time (41). Personnel travel time and travel-related operating costs were considered assuming an average travel distance of 50 kilometer (km) per patient (4, 38) and a flat reimbursement rate of 0.30€ per km (42). Pharmaceutical and medical supply costs are assumed to be comparable to inpatient treatment and are modeled accordingly using the InEK cost matrix. Medical and non-medical infrastructure costs (e.g., administration, information technology, office space, staff training) were estimated by applying a 27% overhead surcharge to personnel and material costs, based on the InEK cost matrix and recommendations for non-valuated PEPP (43). In total, estimated costs amounted to 324.11€ per treatment day. The daily costs were validated against unpublished institutional data from a German psychiatric hospital, which reported regionally negotiated flat rates ranging from 315€ to 360€ per patient-day. This is also consistent with the cost inputs applied in comparable StäB evaluations in the literature (38).

We assumed that all patients receive outpatient psychotherapeutic follow-up treatment from outpatient therapists following the acute treatment phase. This assumption serves to standardize cost estimation, although it does not reflect the full variability of real-world treatment. In the base-case analysis, an intermediate frequency of one session every two weeks was considered. The cost per session is estimated using item 35,425 of the German Uniform Value Scale (Einheitlicher Bewertungsmaßstab), which refers to 50-minute individual cognitive-behavioral therapy, with an approximate reimbursement rate of 116€ per session (44). Outpatient follow-up costs are applied equally to both intervention strategies (inpatient vs. home treatment). This is based on evidence from studies included in Foremnik et al. which found no significant differences in the frequency of outpatient psychotherapeutic utilization between groups (11, 12, 18). No costs are assigned to the health state “death”.

### 2.6 Sensitivity analyses

To assess the robustness of model results under parameter uncertainty, both deterministic (one-way) and probabilistic sensitivity analyses were conducted. All parameter ranges are reported in Table 1.

For readmission probabilities, uncertainty ranges were derived using the Wilson score confidence interval for binomial parameters, calculated from reported sample proportions and sample sizes (45). As baseline readmission rates reported by Graf et al. were already at the upper range of estimates of published studies (15, 29, 30), only the lower bound of the confidence interval was applied (18). For remission probabilities, 95% confidence intervals were calculated from the standard deviation and sample size reported by Graf et al. (18) Suicide-related transition probabilities were varied using the 95% confidence intervals reported by Chung et al., converted into monthly transition probabilities (19).

For utility values, *a* ± 10% range was applied around the base-case estimates. As utility values were equated between groups in the base case and empirical standard deviations exceeded plausible bounds, this range was considered the most appropriate approach to characterizing uncertainty.

For inpatient costs, the least and most expensive PEPP categories within the applicable diagnosis group were used as lower and upper bounds, respectively.

For home treatment costs, parameters were varied individually. Treatment intensity was varied between one and two daily patient contacts, with profession-specific scenarios reflecting either a medical or a nursing-led staffing composition. The multiplier for indirect personnel time was varied by ±1 standard deviation around the base-case value of 0.33 (41). Travel distance was varied by ±15 km around the base-case assumption of 50 km (46); the lower bound reflects the potential for cost-efficient routing in practice. The overhead surcharge was varied between 20% (11) and 31%, reflecting the cost share of the most expensive PEPP in the InEK cost matrix, with the base case set at 27%.

Probabilistic sensitivity analyses were conducted using Monte Carlo simulation with 10,000 iterations to simultaneously propagate uncertainty across all model parameters in AMUA (47). Beta distributions were assigned to transition probabilities and utility values, while gamma distributions were used for cost parameters.

## 3 Results

**Figure 2:**
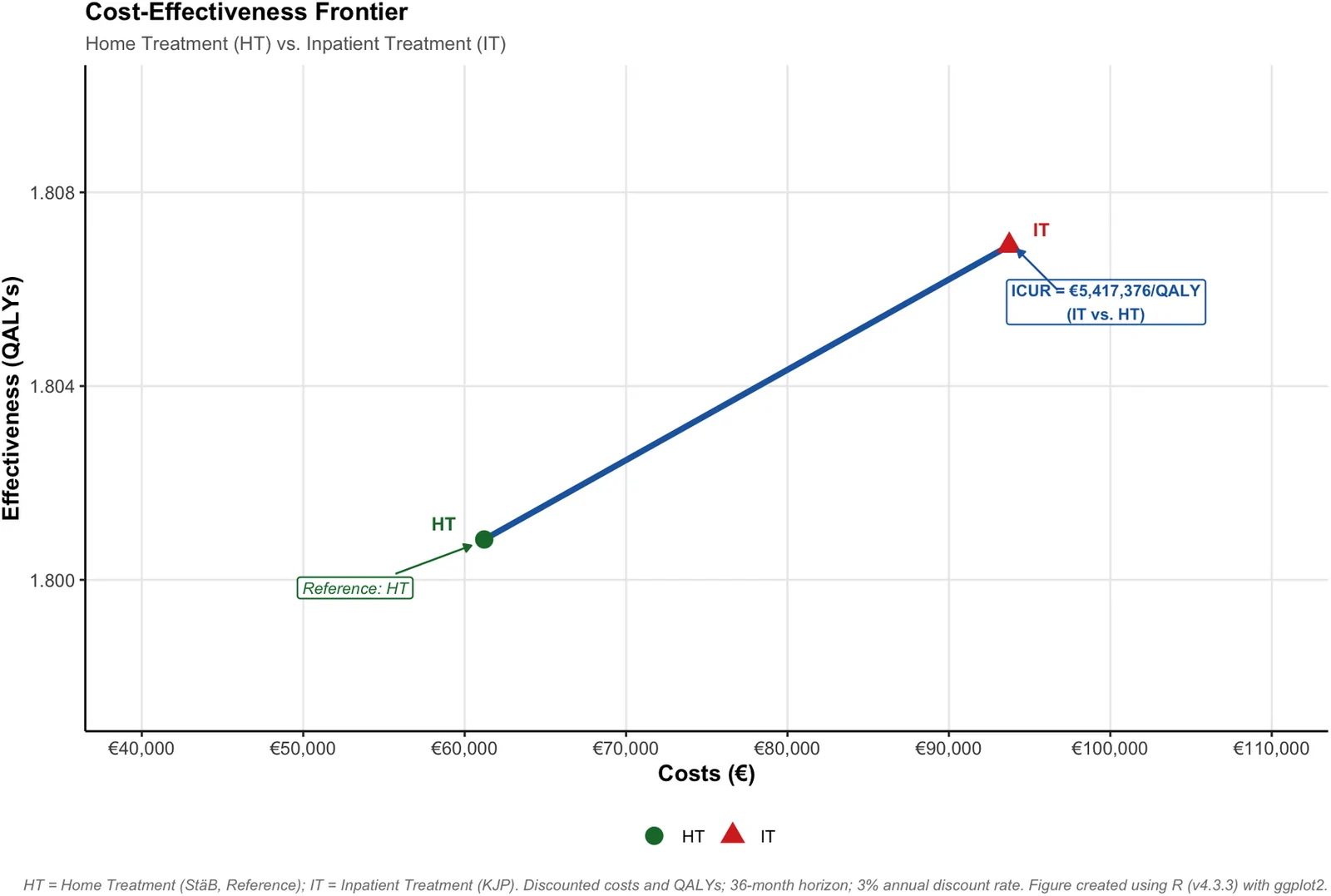
Cost-Effectiveness frontier.

In the base-case analysis, home treatment was less costly compared to inpatient treatment and slightly less effective compared to inpatient treatment. Switching from home treatment to inpatient treatment would yield 0.006 QALYs gained and incremental costs of 32,524€ resulting in an ICUR of 5,417,376€/QALY gained. (Table 2 and Figure 2).

**Table 2:** Base-case analysis: absolute and incremental discounted QALYs, costs and incremental cost-utility ratios for inpatient (IT) and home treatment (HT) approaches.

Strategy	Total Costs (€)	Total QALYs	*Δ*Costs (€)	*Δ*QALY	ICUR (€/QALY)
HT	61,213	1.801	—	—	—
IT	93,737	1.807	32,524	0.006	5,417,376

HT, home treatment; ICUR, incremental cost-utility ratio; IT, inpatient treatment; QALY, quality-adjusted life years.

Costs and effects were discounted at 3% per year.

Results were generally robust in one-way and probabilistic sensitivity analyses. One-way sensitivity analysis based on incremental net monetary benefit (INMB, at a WTP threshold of 90,000€/QALY, with HT as the reference strategy), confirmed that HT remained the cost-effective strategy across nearly the entire tested parameter range (base-case INMB =  + 31,983€). Only variation in remission probability was identified as the most influential and uncertain parameter in the one-way sensitivity analysis: INMB ranged from −65,698€ to +26,239€ when varying the monthly remission probability for HT (base case: 0.302; range: 0.273–0.339). INMB ranged from −68,044€ to +11,560€ when varying the monthly remission probability for IT (base case: 0.282; range: 0.201–0.520) (see Figure 1 in Supplementary Material).

The probabilistic sensitivity analysis, supported the base-case results, indicating that inpatient treatment was more costly and only marginally more effective than home treatment. Accordingly, 87% of the probabilistic simulations were in the northeast quadrant of the cost-effectiveness plane (higher costs and higher effects for IT compared with HT) (see Figure 2a in Supplementary Material). Given the WTP threshold of 90,000€ per QALY, the probability that IT was cost-effective compared with HT was only 0.6% see Figure 2a in Supplementary Material).

## 4 Discussion

This is the first Markov model-based cost-utility analysis comparing HT with IT for children and adolescents experiencing acute psychiatric crises. The base-case analysis found HT to be associated with cost savings of 32,524 € per patient over a three-year time horizon, alongside a marginal and non-significant QALY loss of 0.006. The resulting ICUR of 5,417,376€/QALY suggest that HT may represent a cost-effective alternative to IT under the assumptions of the present model. However, given the limited evidence base underlying the model parameters, these findings should be interpreted with caution, as they depend on assumptions that remain to be validated using more robust empirical data.

These findings align with and extend the limited prior economic evidence in child and adolescent psychiatry. Boege et al. reported cost savings alongside superior clinical outcomes for the sequential HoT-BITs model, suggesting dominance of HT (11). Ougrin et al., evaluating the SDS model, found only a moderate probability of economic viability (approximately 58%–60%) (12). Evidence from multisystemic therapy (MST) further suggests short-term cost advantages over inpatient treatment, though these attenuate over a 12-month follow-up, with costs and outcomes converging between treatment arms (48). The considerably stronger cost-effectiveness signal observed in the present analysis is primarily driven by the large cost differential between StäB-based HT (approximately 324€/day) and inpatient treatment (approximately 584€/day), which substantially outweighs the marginal QALY disadvantage observed in the HT group. Although QALYs were assumed equivalent across settings, higher readmission rates in the HT group suggest outcomes may marginally favor IT.

Notably, the finding of higher readmission rates under HT- albeit not statistically significant (9, 10)- appears to contradict the hypothesis that HT would reduce crisis recurrence through more sustainable treatment effects. HT models may facilitate earlier and more frequent recontacts with services before a full crisis develops, potentially preventing longer and more costly inpatient admissions. Whether the higher readmission rates in the HT group reflect a genuine increase in relapse risk or rather reflect a lower threshold for treatment entry remains an open empirical question that future studies should address.

Health-related quality of life, as measured by the EQ-5D-3L, did not differ significantly between treatment groups in the only identified study reporting this outcome, with follow-up of six months post-randomization (12). Utility values were therefore equated across treatment arms in the base case. These values were derived from a UK source (12), as no comparable German estimates were available.

The use of the EQ-5D-3L in this context warrants critical discussion. First, the instrument was not designed for use in children and adolescents; condition-specific alternatives such as the Child Health Utility 9D (CHU9D) or the EQ-5D-Y should be considered in future trials (49)) Second, the EQ-5D-3L captures primarily generic health dimensions and may inadequately reflect aspects of quality of life that are particularly salient for individuals with mental health conditions. Qualitative evidence suggests that dimensions such as hope, autonomy, sense of belonging, and self-perception are central to quality of life in this population- none of which are covered by the EQ-5D (50). This is especially relevant in the context of home treatment, which may specifically affect such social and relational domains: Evidence from meta-analytic data suggests that home treatment may produce more sustained improvements in youth social functioning compared to IT (10).

Against this background, Sen's Capability Approach offers a potentially valuable theoretical framework for incorporating broader outcome dimensions into health economic evaluations. Rather than asking solely how healthy a person is, this approach asks whether individuals have the opportunity to do and be what they have reason to value (51, 52). Capability-based instruments may therefore serve as a meaningful complement to conventional QALY-based measures, though their application in children and adolescents presents methodological challenges, given that autonomy, decision-making capacity, and future opportunities are still developing across this age range (53).

Finally, it should be noted that, unlike the United Kingdom and several other OECD countries, German health policy decision-makers do not formally accept WTP thresholds for QALY gains as a basis for resource allocation (22, 54, 55). Nevertheless, the QALY framework remains the most widely applied methodology in health economic evaluations, providing a theoretically grounded, preference-weighted measure of treatment effectiveness (22). Known limitations- including the lack of a universally accepted definition of perfect health and concerns regarding the undervaluation of health states in disabled or chronically ill populations (56) should nonetheless be acknowledged when interpreting QALY-based results. Improvements in these domains may therefore go undetected, potentially leading to a systematic underestimation of treatment benefit in psychiatric populations. Several limitations of the present analysis should be acknowledged.

First, a major limitation relates to the availability and quality of input data. Transition probabilities for readmission and remission were derived from a single non-randomized study with a relatively small sample size of 75 patients in total (18). The retrospective, non-randomized design of this primary data source constitutes an additional limitation, as treatment allocation to HT vs. IT was choice-based and patients with more severe psychiatric conditions may have been preferentially assigned to IT, potentially introducing selection bias. Moreover, data collection during the COVID-19 pandemic may have inflated readmission rates in the study, limiting the generalizability of findings to non-pandemic settings (18). Model parameters were derived from different studies. Consequently, the underlying study populations varied with respect to age distribution, diagnostic composition, and country. These differences may limit the internal consistency and generalizability of the model inputs. Each source was selected because it provided the most valid evidence for the respective parameters. In addition, these parameters were varied in sensitivity analyses to assess their impact on results.

A second limitation concerns the operationalization of remission. In the absence of a validated transdiagnostic definition of remission applicable to children and adolescents presenting with heterogeneous acute psychiatric crises, remission was pragmatically defined as discharge from hospital-equivalent treatment, with length of stay used as a proxy. However, discharge does not necessarily correspond to clinical remission. Patients may be discharged for reasons unrelated to full clinical recovery, including system-level pressures such as limited bed capacity, institutional discharge policies, or service organization constraints. This distinction is particularly relevant when interpreting findings across healthcare systems, as discharge thresholds and treatment pathways may vary substantially between countries and clinical settings.

The third structural limitation is the assumption that patients remain within their initial treatment arm throughout the model. Relapses in the home treatment arm were therefore modelled as re-entries into home treatment, while relapses in the inpatient arm were modelled as re-admissions to inpatient treatment. In practice, however, treatment pathways are more fluid: patients may switch between settings, receive intermittent inpatient treatment during otherwise home-based episodes, or be assigned to a different treatment modality in subsequent crises. This assumption may partly explain the robust results observed in the present analysis, as the full cost differential between StäB-based HT and IT was applied consistently across the entire time horizon. Evidence from adult psychiatry indicates that HT may shift costs from inpatient to outpatient sectors, while total costs across sectors may converge between treatment arms (55). If comparable cost-shifting occurs in child and adolescent psychiatry, the present model may overestimate the economic advantage of HT. Moreover, recurrent episodes were assumed to occur under the same clinical and economic conditions as the index episode, although in practice subsequent episodes may differ in treatment allocation, intensity, duration, and associated costs.

Fourth, the substantial heterogeneity of HT approaches limits the transferability of findings across settings. Models vary in staffing composition, contact frequency, treatment intensity, duration, and whether they are delivered as stand-alone or sequential interventions. This distinction was also relevant for parameter selection in the present analysis: transition probabilities and costs were based on a study incorporating a stand-alone HT approach matching the intervention of this decision-analytic study. Whereas utility values were derived from a study evaluating a sequential treatment approach due to lack of utility data specific for stand-alone HT were available (12). Such variation is likely to influence both costs and outcomes: greater treatment intensity may increase costs but also improve clinical effectiveness, reduce relapse risk, and prevent costly inpatient admissions. Accordingly, the present findings should be interpreted within the specific regulatory, reimbursement, and organizational context of the German StäB model.

This analysis highlights several priorities for future research. Most urgently, randomized controlled trials with longer follow-up periods are needed to provide robust estimates of readmission rates and time-to-event data, ideally reported using Kaplan–Meier methods stratified by treatment arm. Future trials should incorporate health-related quality of life assessment using instruments specifically validated for children and adolescents.

Studies should also distinguish clearly between stand-alone and sequential home treatment models, as these may differ substantially in their cost and effectiveness profiles. In addition, partial hospitalization or day-hospital care should be considered as a relevant alternative treatment setting. Future studies should directly compare the cost-effectiveness of home treatment, inpatient treatment, and day-hospital care. Future analyses should also adopt a broader societal perspective, as HT is delivered within the family environment and may require greater caregiver involvement, exclusion of indirect costs such as parental productivity losses may lead to an underestimation of the total burden.

From a health services planning perspective, the present analysis provides preliminary support for the cost-utility of HT as a daily-contact outreach service for children and adolescents in acute psychiatric crisis. While resource allocation decisions should be made cautiously given the scarcity of high-quality evidence, the findings suggest that HT may remain cost-effective over a longer time horizon and may represent a viable alternative to IT for selected patients.

## Data Availability

The original contributions presented in the study are included in the article/Supplementary Material, further inquiries can be directed to the corresponding author/s.
