# Supplementary material for: Cost-utility analysis of home treatment versus inpatient treatment in child and adolescent psychiatry: a decision analysis
Source: Front Child Adolesc Psychiatry. 2026 Aug 10;5:1923381. doi: 10.3389/frcha.2026.1923381 (PMC13500286; doi:10.3389/frcha.2026.1923381)
Supplement: Supplementary file 1 [file Datasheet2.pdf]

CHEERS 2022 Checklist

**Title**			
Title	1	Identify the study as an economic evaluation and specify the interventions being compared.	Title, Page 1
**Abstract**			
Abstract	2	Provide a structured summary that highlights context, key methods, results, and alternative analyses.	Abstract, Page 1-2
**Introduction**			
Background and objectives	3	Give the context for the study, the study question, and its practical relevance for decision making in policy or practice.	Introduction, Line 89-94
Methods			
**Health economic analysis plan**	**4**	**Indicate whether a health economic analysis plan was developed and where available.**	**not available**
Study population	5	Describe characteristics of the study population (such as age range, demographics, socioeconomic, or clinical characteristics).	Analytical Framework , Line 143-150
Setting and location	6	Provide relevant contextual information that may influence findings.	Analytical Framework , Line 156-161
Comparators	7	Describe the interventions or strategies being compared and why chosen.	Analytical Framework , Line 137-142
Perspective	8	State the perspective(s) adopted by the study and why chosen.	Analytical Framework , Line 156-161
Time horizon	9	State the time horizon for the study and why appropriate.	Analytical Framework , Line 151-155

(continued)

Discount rate	10		Report the discount rate(s) and reason chosen.	Analytical Framework , Line 154-155
Selection of outcomes	11		Describe what outcomes were used as the measure(s) of benefit(s) and harm(s).	Analytical Framework , Line 162-171
Measurement of outcomes	12		Describe how outcomes used to capture benefit(s) and harm(s) were measured.	Analytical Framework , Line 162-171
Valuation of outcomes	13		Describe the population and methods used to measure and value outcomes.	Measurement of Utilities
Measurement and valuation of resources and costs	14		Describe how costs were valued.	Measurement of Costs
Currency, price date, and conversion	15		Report the dates of the estimated resource quantities and unit costs, plus the currency and year of conversion.	Measurement of Costs , Line 232-234
Rationale and description of model	16		If modelling is used, describe in detail and why used. Report if the model is publicly available and where it can be accessed.	Introduction, Line 73-77
Analytics and assumptions	17		Describe any methods for analysing or statistically transforming data, any extrapolation methods, and approaches for validating any model used.	Transition probabilities, Line 185-196, 206-213
Characterising heterogeneity	18		Describe any methods used for estimating how the results of the study vary for subgroups.	Discussion
Characterising distributional effects	19		Describe how impacts are distributed across different individuals or adjustments made to reflect priority populations.	not reported

(continued)

Characterising uncertainty	20	Describe methods to characterise any sources of uncertainty in the analysis.	Results, Line 336-355; Fig.2+3
Approach to engagement with patients and others affected by the study	21	Describe any approaches to engage patients or service recipients, the general public, communities, or stakeholders (such as clinicians or payers) in the design of the study.	not reported
**Results**			
Study parameters	22	Report all analytic inputs (such as values, ranges, references) including uncertainty or distributional assumptions.	Table 1
Summary of main results	23	Report the mean values for the main categories of costs and outcomes of interest and summarise them in the most appropriate overall measure.	Results, Line 329-335, Figure 3
Effect of uncertainty	24	Describe how uncertainty about analytic judgments, inputs, or projections affect findings. Report the effect of choice of discount rate and time horizon, if applicable.	Results, Line 336- 340, Figure 2
Effect of engagement with patients and others affected by the study	25	Report on any difference patient/service recipient, general public, community, or stakeholder involvement made to the approach or findings of the study	Not reported
Discussion			

(continued)

Study findings, limitations, generalisability, and current knowledge	26	Report key findings, limitations, ethical or equity considerations not captured, and how these could affect patients, policy, or practice.	Discussion
Other relevant information			
Source of funding	27	Describe how the study was funded and any role of the funder in the identification, design, conduct, and reporting of the analysis	End of manuscript
Conflicts of interest	28	Report authors conflicts of interest according to journal or International Committee of Medical Journal Editors requirements.	End of manuscript

From: Husereau D, Drummond M, Augustovski F, et al. Consolidated Health Economic Evaluation Reporting Standards 2022 (CHEERS 2022) Explanation and Elaboration: A Report of the ISPOR CHEERS II Good Practices Task Force. Value Health 2022;25. doi:10.1016/j.jval.2021.10.008
