# Supplementary material for: Cost-utility analysis of home treatment versus inpatient treatment in child and adolescent psychiatry: a decision analysis
Source: Front Child Adolesc Psychiatry. 2026 Aug 10;5:1923381. doi: 10.3389/frcha.2026.1923381 (PMC13500286; doi:10.3389/frcha.2026.1923381)
Supplement: Supplementary file 2 [file Datasheet3.pdf]

Supplementary Material: Sensitivity Analyses

For the deterministic sensitivity analysis, the incremental net monetary benefit (INMB) was used as the outcome measure and visualized in a tornado diagram. This approach was chosen because the very small incremental QALY difference between inpatient treatment (IT) and home treatment (HT) can lead to unstable or undefined ICUR estimates. INMB was calculated as NMB_IT minus NMB_HT at a willingness-to-pay threshold of 90,000€ per QALY. Therefore, negative INMB values indicate that HT remains preferred, whereas positive INMB values would favour IT. Overall, the deterministic sensitivity analysis supported the robustness of the base-case result, with HT remaining the preferred strategy across most parameter variations. However, the result was sensitive to assumptions regarding remission probabilities, particularly the remission probability for HT.

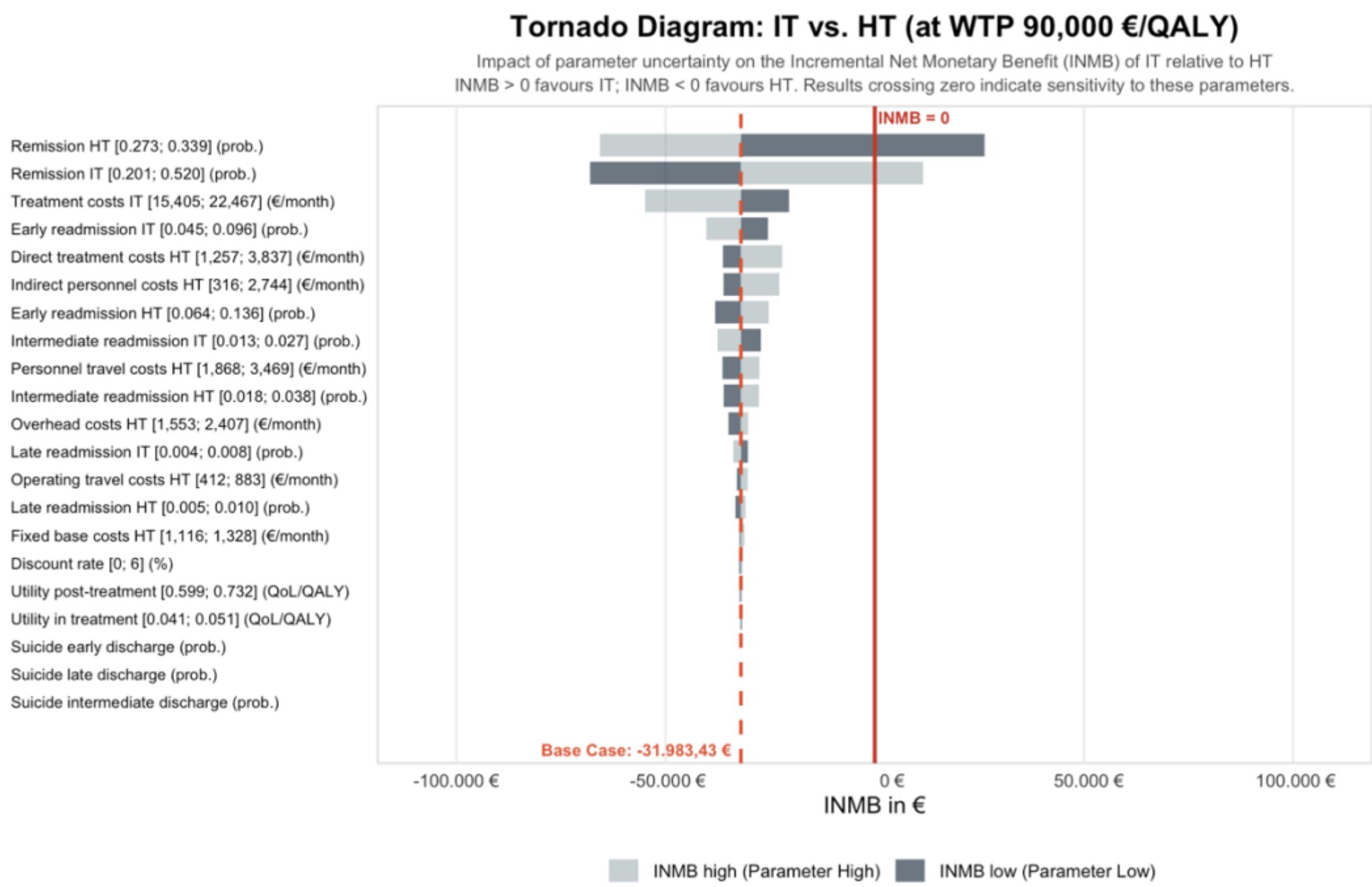

Figure 1. Results of the deterministic sensitivity analysis

In the probabilistic sensitivity analysis, based on 10,000 Monte Carlo simulations, inpatient treatment (IT) was more costly and slightly more effective than home treatment (HT). Accordingly, most probabilistic simulations were in the northeast quadrant of the cost-effectiveness plane, indicating higher costs and higher effects for IT compared with HT (Figure 2a). Switching from HT to IT resulted in an incremental gain of 0.006 QALYs and additional costs of 32,524€, corresponding to an ICUR of more than €5 million per QALY gained. Given the willingness-to-pay threshold of 90,000€ per QALY, the probability that IT was cost-effective compared with HT was only 0.6% (Figure 2b). Thus, HT remained the preferred reference strategy in the probabilistic analysis.

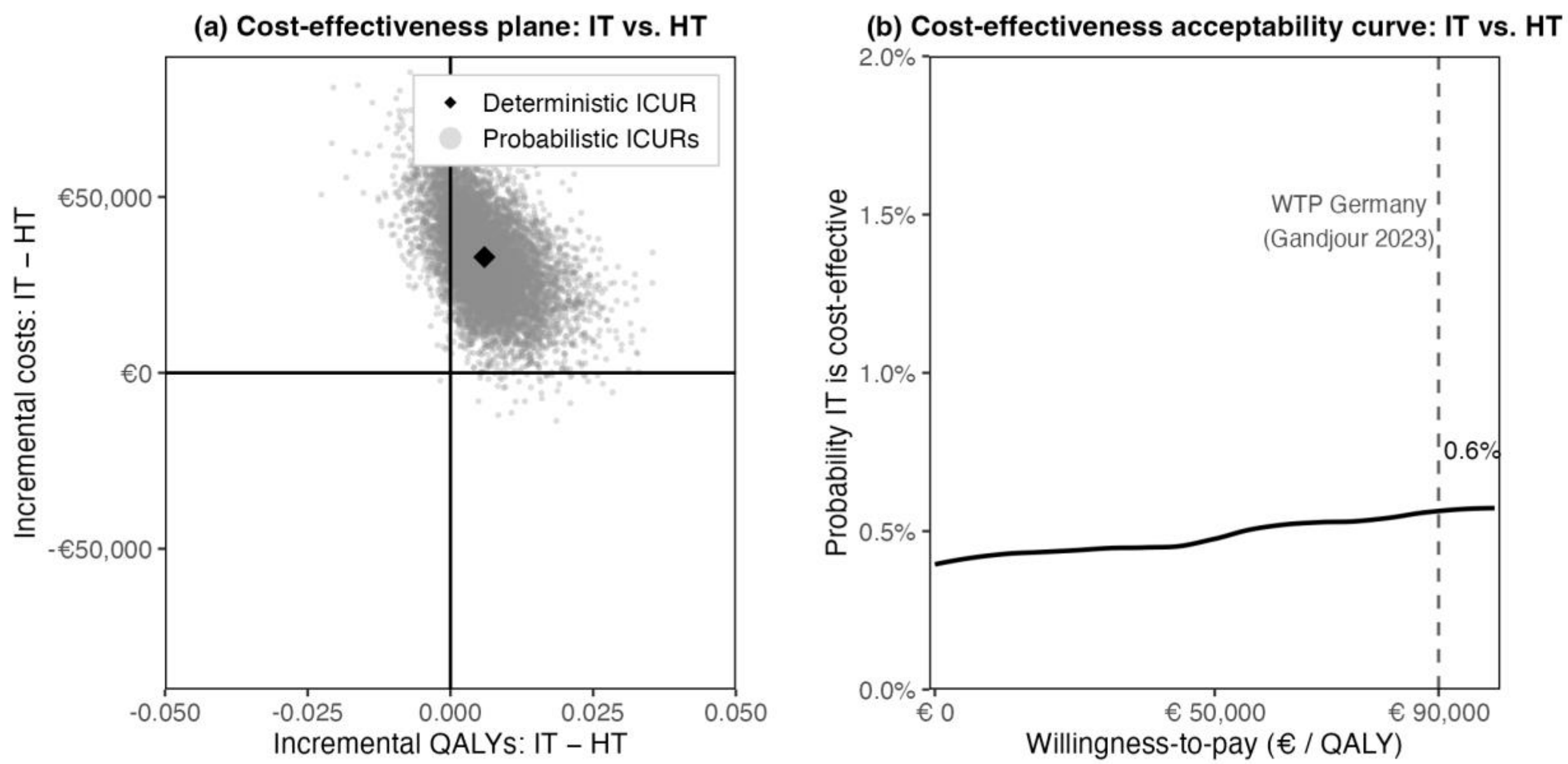

Figure 2. Results of the probabilistic sensitivity analysis
