# Supplementary material for: Cost-utility analysis of home treatment versus inpatient treatment in child and adolescent psychiatry: a decision analysis
Source: Front Child Adolesc Psychiatry. 2026 Aug 10;5:1923381. doi: 10.3389/frcha.2026.1923381 (PMC13500286; doi:10.3389/frcha.2026.1923381)
Supplement: Supplementary file 3 [file Datasheet4.pdf]

Supplementary Material: Population and study characteristics

Study	Model parameter informed	Country/ setting	Study design	Sample	Age	Sex	Diagnoses/clinical characteristics		Follow-up
Graf et al. 2025	Transition probabilities (Readmissions, treatment duration)	Switzerland	CBA (preference-based group allocation)	75 (IG: 27; CG: 48)	6-17 yrs. (M = 15.92 yrs., SD = 2.87)	IG: 48% female; CG: 69% female	Adolescents with acute mental disorders (affective, anxiety, behavioral).	AT_Home=stand-alone model (avg. 84 days; 1×/d (60–120 min); 24/7 crisis line) vs. Inpatient treatment (no time limit; avg. 101 days)	18-24 months post-treatment
Ougrin et al. 2018	Health state utilities (EQ-5D-3L)	United Kingdom	RCT	106 (IG: 53; CG: 53)	12-17 yrs. (IG: M= 16.23 yrs., SD= 1.54; CG: M= 16.34 yrs., SD= 1.7)	IG: 68% female; CG: 62% female	Adolescents presenting with psychiatric emergencies/ all psychiatric illnesses warranting inpatient care; no exclusion based solely on risk level (if risk to self or others was deemed manageable).	SDS/ ICCS (Early inpatient discharge (median 34 d), followed by flexible SDS/ ICCS home/day care (mean total 116.3 d); daily contact possible; available 8–20 h + 24/7 on-call) vs. Inpatient treatment (median 50 days) followed by CAMHS outpatient treatment)	6 months after randomization
Chung et al. 2017	Post-discharge suicide mortality	Multinational: 100 studies pooled from Asia, Australasia, mainland Europe, Nordic countries, North America, UK/Ireland (high-income countries only)	Systematic review and meta-analysis of observational cohort studies	100 studies, 183 samples overall; adol escent subgroup: 20 of 183 samples (discharge age ≤18 years)	Pooled analysis stratified by age group at discharge: adolescents (≤18 yrs), adults/unspecified, older patients (≥65 yrs); no mean/SD reported (categorical subgroup only)	Overall sample: 50 female-only, 49 male-only, 84 mixed-sex samples	Heterogeneous discharged psychiatric inpatients (psychosis, affective disorders, mixed/other diagnoses); includes subgroup admitted specifically for suicidality; no diagnosis-based restriction	Not applicable; mortality estimates were not specific to a particular intervention or comparator	Highly variable across included studies (categorized as 0–3 mo, >3–12 mo, 1–5 y, 5–10 y, >10 y); overall median follow-up 60 months (range 1–432 months)

Abbreviations: AT_HOME, home treatment intervention evaluated by Graf et al.; CAMHS, Child and Adolescent Mental Health Services; CG, comparison group; EQ-5D-3L, EuroQol 5-Dimension 3-Level questionnaire; ICCS, Intensive Community Care Service; IG, intervention group; M, mean; N, total sample size; n, subgroup sample size; RCT, randomized controlled trial; SD, standard deviation; SDS, Supported Discharge Service.
